# Development of Activity-Related Muscle Fatigue during Robot-Mediated Upper Limb Rehabilitation Training in Persons with Multiple Sclerosis: A Pilot Trial

**DOI:** 10.1155/2015/650431

**Published:** 2015-05-24

**Authors:** Johanna Renny Octavia, Peter Feys, Karin Coninx

**Affiliations:** ^1^Parahyangan Catholic University, Industrial Engineering Department, Ciumbuleuit 94, Bandung 40141, Indonesia; ^2^Hasselt University, Expertise Centre for Digital Media-tUL-iMinds, Wetenschapspark 2, 3590 Diepenbeek, Belgium; ^3^REVAL Rehabilitation Research Center, BIOMED, Faculty of Medicine and Life Sciences, Hasselt University, Agoralaan Gebouw A, 3590 Diepenbeek, Belgium

## Abstract

Robot-assisted rehabilitation facilitates high-intensity training of the impaired upper limb in neurological rehabilitation. It has been clinically observed that persons with Multiple Sclerosis (MS) have difficulties in sustaining the training intensity during a session due to the development of activity-related muscle fatigue. An experimental observational pilot study was conducted to examine whether or not the muscle fatigue develops in MS patients during one session of robot-assisted training within a virtual learning environment. Six MS patients with upper limb impairment (motricity index ranging from 50 to 91/100) and six healthy persons completed five training bouts of three minutes each performing lifting tasks, while EMG signals of anterior deltoid and lower trapezius muscles were measured and their subjective perceptions on muscle fatigue were registered. Decreased performance and higher subjective fatigue perception were present in the MS group. Increased mean EMG amplitudes and subjective perception levels on muscle fatigue were observed in both groups. Muscle fatigue development during 15′ training has been demonstrated in the arm of MS patients, which influences the sustainability of training intensity in MS patients. To optimize the training performance, adaptivity based on the detection of MS patient's muscle fatigue could be provided by means of training program adjustment.

## 1. Background

Multiple Sclerosis (MS) is a chronic progressive disease while to date, no medicine has been found to cure people suffering from MS yet. Thus, the aim of therapy and rehabilitation for MS patients is to maintain and even improve their functional mobility by reducing disuse and dependency and improve quality of life. In the past, intensive physical training was not advised for MS patients due to the opinion that it would advance the deterioration by means of relapses, which appeared untrue [1]. Now however, performing physical training is often part of therapy and rehabilitation for MS patients [2]. A number of studies have shown beneficial effects of physical training in MS regarding muscle strength, exercise tolerance, functional mobility (i.e., walking), and quality of life, while no harmful effects were reported [3–6].

Upper limb rehabilitation is considered important since upper limb impairments, prevalent in MS especially at an advanced stage, strongly influence the capacity of MS patients to perform several activities of daily living (ADL) such as self-feeding (e.g., eating, drinking, and taking medications), personal care (e.g., bathing, brushing hair), and dressing [7]. Generally in a conventional rehabilitation training session with a therapist, only a limited time is dedicated to upper limb training given the presence of a multiplicity of other disabling symptoms in the advanced stage of MS. Although traditional rehabilitation methods may have the potential to improve upper limb function [8], it is considered that additional therapeutic modalities are necessary to enable MS patients to train independently of the therapist and more intensively.

Robot and sensor technologies are considered to be promising to provide an effective and independent upper limb rehabilitation training in stroke in order to improve motor control while also indications of improving daily life activities are present [9–11]. Robot-mediated rehabilitation allows high-intensity, repetitive, task-specific, interactive treatment of the impaired upper limb which are considered as vital factors contributing to a successful neurological rehabilitation [12, 13]. Also in MS, studies have been published on the integration of robot-assisted rehabilitation into the rehabilitation training of MS patients to enhance upper limb function [14–16].

In the context of a European interregional research projects (INTERREG-IV projects “Rehabilitation robotics II” and “I-TRAVLE”), the effects of robot-assisted upper limb rehabilitation training for MS patients were investigated. A complete haptic-based rehabilitation system, I-TRAVLE, was developed to support a systematic and personalized upper limb rehabilitation training for MS patients. I-TRAVLE (Individualized, Technology-supported and Robot-Assisted Virtual Learning Environments) utilizes two technologies: robot-assisted rehabilitation and virtual environments [17].

However in MS, we regularly observed cases in which patients, during the upper limb rehabilitation training with I-TRAVLE, were incapable of sustaining the training endurance even during a single session of 15 minutes. The high repetitiveness of the training exercises in a virtual environment tends to make patients tired after training for a while but they also experienced increasing difficulties during performing the training exercises on their own. We also noticed compensatory movements in the shoulder, neck, and trunk of some patients during training, which might cause adverse events as muscle soreness and musculoskeletal disorders in the long term. These observations are likely related to fatigability, also called muscle or motor fatigue, that is prevalent in persons with MS both for upper and lower limb isolated sustained or repeated muscle contractions [18–22]. Fatigability refers to a magnitude or rate of change in a performance criterion relative to a reference value over a given time of a task performance or measure of mechanical output [23]. Besides, fatigability can also be indicated by increased EMG amplitudes, which has been widely accepted as a valuable tool to indicate muscle fatigue, measured over a period of time [24–26].

A possible solution to this phenomenon is to provide the patients with some assistance during the training to help them perform the exercise and sustain the training endurance which is important from a neural drive point of view. Such assistance can be gravity compensation of the arm, which is shown to provide support and be beneficial for the quality and active range of upper limb movement of MS (and other neurological) patients during their upper limb rehabilitation [16, 27, 28]. The level of such assistance is usually determined based on the muscle strength of the patient and only once at the beginning of the training. However during the course of the training, this assistance level might be insufficient to help patients sustain the training endurance without detrimental compensatory movements or prevent their muscle from abnormal fatiguing. The longer they train, the more tiring they become, and thus more assistance might be necessary to keep up their performance and motivation.

The present study aims to investigate the development of muscle fatigue in MS patients during a session of robot-mediated upper limb rehabilitation training. We describe an experiment in MS patients during their rehabilitation training with I-TRAVLE, conducted to observe the development of muscle fatigue, whether or not adaptive additional movement assistance is indicated in future developments. We utilized electromyography (EMG) signals to measure the muscle activity and determine the muscle fatigue in MS patients. The utilization of EMG to measure muscular effort in MS patients has been previously applied [24] and has been widely accepted as a valuable tool to indicate muscle fatigue [25, 26]. Besides that, we measured game performance and subjective fatigue levels during each 3-minute lasting training bouts, accumulating to a 15-minute training session.

## 2. Methods

### 2.1. Participants

Twelve participants were recruited to take part in the experiment. They were six patients of the Rehabilitation and MS Centre in Overpelt (Belgium) who all suffer from muscle weakness due to MS, and another six age- and sex-matched healthy persons who were selected to participate as a control group. The healthy participants were 5 males and 1 female, with the mean age of 59 ± 9.6 (range 43 to 70 years). The MS patients were 5 males and 1 female, with the mean age of 58.2 ± 7.5 (range 45 to 66 years). The mean duration of the MS diagnosis was 16.2 ± 12.7 (range 6 to 36 years). To document the severity of their upper limb dysfunction, we applied clinical measures for upper limb strength (motricity index [29]) and arm motor function tests (Brunnstrom Fugl-Meyer proximal and distal [30]).  Table 1 gives an overview of the personal information and clinical measures of MS patients participating in this experiment.

Prior to the experiment, approval on the study was acquired from the local ethics committee and signed informed consent was obtained from all participants.

### 2.2. Apparatus

**Figure 1:**
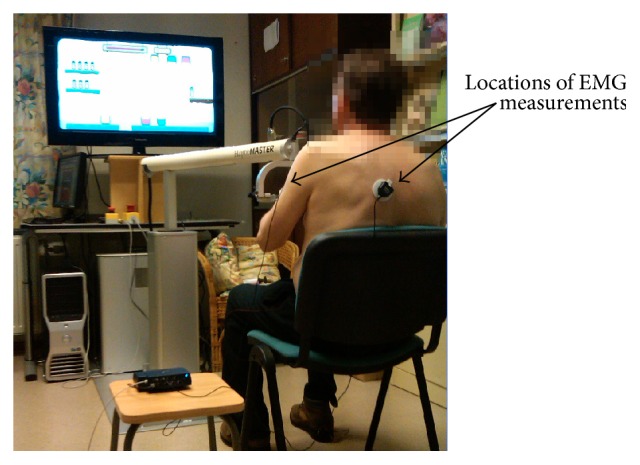
Experimental apparatus and setup.

We used the I-TRAVLE system with the HapticMaster for this experiment, which are partially described previously [31]. The system consists of a hardware and software system setup as depicted in Figure 1, which also illustrates the experimental apparatus and setup.

**Figure 2:**
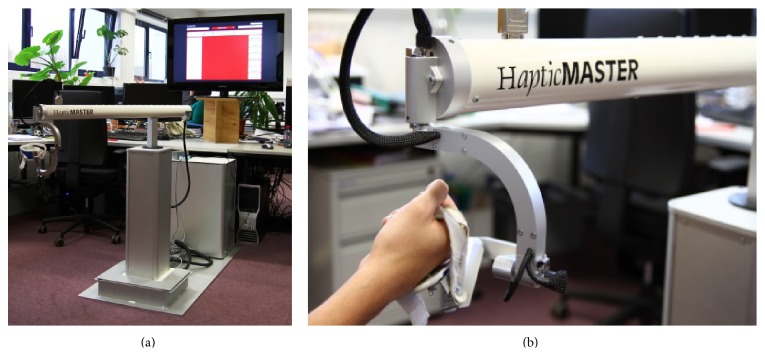
I-TRAVLE hardware system: MOOG HapticMaster and visual display (a), ADL gimbal (b).

The main component of the hardware system is a haptic robot, the MOOG HapticMaster as illustrated in Figure 2, which functions as both input and output devices. As an input device, it allows patients to interact with the software applications that deliver the training exercises by detecting the patient's movements to be displayed in the virtual learning environment. As an output device, it provides haptic feedback during the training by guiding or hindering with exerted forces. The HapticMaster is equipped with a peripheral device, the ADL gimbal, where the patients' hand is placed and secured using the attached brace while performing the training exercises. The gimbal attachment allows the patient to make unrestricted wrist and hand movements. A large display, a full HD 40′′ Samsung TV screen, is used as a visual display to project the training exercises and is placed behind the HapticMaster approximately 1.5 m in front of the patient.

The main components of our I-TRAVLE software system are the training exercises, the patient interface, the therapist interface, and the central database. Within I-TRAVLE, the training exercises are to be performed in a virtual environment and were designed based on those skill components that individual patients need to train related to their upper limb dysfunction. Two types of training exercises were provided, namely, basic training exercises which focus on one specific skill component such as lifting, reaching, and turning, and advanced training exercises which combine multiple skill components and include cognitive loads.

During execution of an experimental playful task (see below), EMG was recorded by the ProComp Infiniti hardware and Biograph software from (Thought Technologies, Canada), a system for real-time computerized biofeedback and physiological data acquisition. EMG signals were captured through the Surface Electromyography sensors (MyoScan-Pro 400), digitized, encoded, and transmitted to a computer running the Biograph software.

### 2.3. Experimental Task

In this experiment, we used a game-like training exercise, namely, the penguin painting exercise, which was developed as part of the training exercises in I-TRAVLE [36]. This exercise was designed to incorporate two skill components: lifting and transporting, which are considered to be among the important skill components to be trained in the upper limb rehabilitation for MS patients and which are part of many activities of daily living [32, 33].

**Figure 3:**
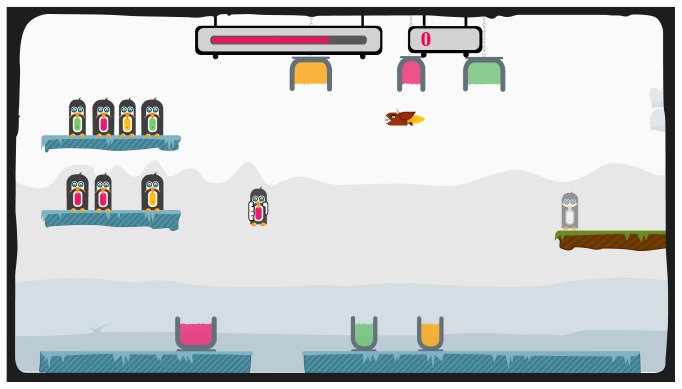
Experimental task: the penguin painting exercise.

The penguin painting exercise is illustrated in Figure 3. The participant has to collect as many points as possible within a certain time period by painting penguins with the matching color. On the left side, there are two shelves with penguins waiting to be painted. The participant has to select one penguin from a shelf and paint it according to the color of its belly. To paint, the participant needs to bring the penguin to the corresponding buckets, by dipping it into the bottom bucket to paint the lower part of the penguin and by sliding into the top bucket for the upper part. While painting, the participant must hold the penguin long enough to effectively apply the color. At some points during the exercise, a devil that tries to capture the penguin appears and must be avoided in order to not lose the penguin already in hand. Every time the participant finishes painting a penguin, the colored penguin must be transported to the exit platform on the right side.

The weight of the penguins was empirically specified to 150 grams for MS participants and 450 grams for the healthy participants with preserved muscle strength. This difference was implemented in order to also induce the process of muscle fatiguing in healthy participants, as they do not have muscle weakness or abnormal fatigue similar to MS participants.

### 2.4. Experimental Procedure

Participants were asked to take place in front of the HapticMaster and were given an explanation on how to use the device and a brief description of the experiment. Two Surface Electromyography sensors were attached to the participants. EMG sensors were attached on the muscles relevant to the lifting movement (the main movement in the penguin painting exercise): the anterior deltoid muscle (as the agonist muscle or prime mover) and the lower trapezius muscle (as the synergist muscle or stabilizer).

Then, participants were asked to manipulate a virtual box shown on the screen in order to determine their active range of motion (AROM). With AROM, we referred to the active workspace in which to perform the training. Participants needed to maximally expand a virtually displayed box in three dimensions, in order to determine their individual active workspace. As such, the I-TRAVLE system can match the individualized performed movement range with the visualization in the virtual movement games. Once determined, participants started with a practice bout of performing the penguin painting exercise for 1 minute to get used to the HapticMaster and game-like task in the virtual environment. After the practice bout, the experiment started. Participants were asked to perform the penguin painting exercise for five consecutive training bouts. Each training bout lasted for 3 minutes. The EMG signals were continuously captured during the training bouts of 15 minutes in total. In between training bouts, patients were asked to rate their subjective fatigue in the arm that was trained (see below). Averagely, the experiment lasted for about 30 minutes per participant.

### 2.5. Outcome Measures and Data Processing

Three outcome measures were collected in the experiment: performance measures, EMG measures, and subjective fatigue measures.

As performance measures, we captured the game scores for each training bout. The game score is determined based on the number of penguins successfully painted and transported to the exit platform in the training environment within the period of 3 minutes. The number of penguins painted is anticipated to decrease as lifting and transporting performance is influenced by fatigue. For each full-painted and transported penguin, the participant receives 2 points. When the penguin is only half-painted, the participant receives 1 point. A total score per training bout of three minutes is provided.

EMG amplitudes have been widely used as one of muscle fatigue indicators [25, 26]. Raw signals from the anterior deltoid and lower trapezius muscles were collected at 2048 Hz and processed in the Biograph software using the Infinite Impulse Response (IIR) filter for the physiological signal [34]. Then, we filtered the data with a Butterworth band pass filter at a defined low cutoff frequency at 0.8 Hz and high cutoff frequency at 2.5 Hz. Since EMG is a bipolar signal, we conducted a rectification process to convert it to a monopolar signal (the positive and negative signals were converted into all positive values). For this purpose, we used the Root Mean Square (RMS) sliding window technique and smoothed the data with an averaging factor of 128 over a time period of 0.125 seconds. Then, we calculated the mean EMG amplitude over each time period of 10 seconds within each training bout. Furthermore, we calculated the increments of the mean EMG amplitudes over the training bouts based on the ratio of the mean EMG amplitude in one training bout compared to the mean EMG amplitude in the previous training bout for each participant.

The subjective perception on muscle fatigue was collected before the start of the experiment and five scores in between each training bout. Participants were asked the following question “*How tiresome does your arm feel at the moment?*” and they had to mark their response on a horizontal line of 10 cm anchored by the word descriptors of* “not tiresome at all”* at the left end and* “very tiresome”* at the right end. The VAS score was determined by measuring the length of the position where the participants have marked their response on the 10 cm horizontal line given. Given the small sample size, we provide mainly descriptive analyses in the results section.

## 3. Results 

### 3.1. Participant's Performance in the Penguin Game

**Figure 4:**
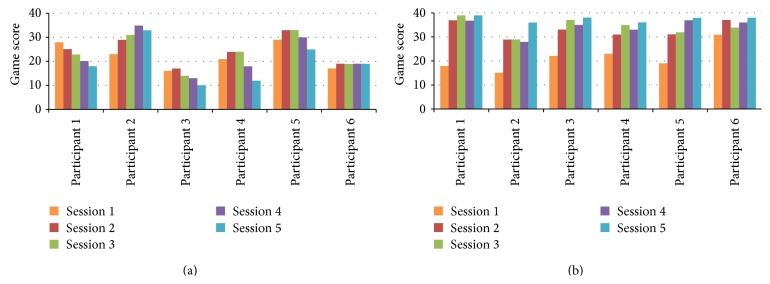
Participant's performance on the penguin game over the 5 training bouts (3′ each): MS participants (a) and healthy participants (b).

Figure 4 shows an overview of the game scores for both MS and healthy participants over the five training bouts. As hypothesized, a decreasing pattern of performance was observed for most of MS participants. Participants MS1 and MS3 showed an immediate performance decrease in the early training bouts, while participants MS4 and MS5 only began to show a performance decrease in the latter training bouts. Participant MS2 showed an increase of performance with a decline in the last training bout. Participant MS6 showed a constant performance over the training bouts. On the other hand, an increasing performance was observed from the healthy participants.

However, Wilcoxon Signed-Rank tests showed that, for MS patients, there is no significant difference in game scores between the first and last bout (*Z* = −1.572, *P* < 0.05). For healthy participants, Wilcoxon Signed-Rank tests showed that there is a significant increase in game scores between the first and last bout (*Z* = −2.201, *P* < 0.05).

Thus, the indication of the muscle fatigue development in both muscles contributing to the lifting movement was supported by the observation of the performance decrease among several MS patients (although not statistically proven), as measured by the game score. However, this finding did not apply to the healthy participants that showed the contrary.

### 3.2. Muscle Fatigue Development during Training Bouts Detected by EMG

**Figure 5:**
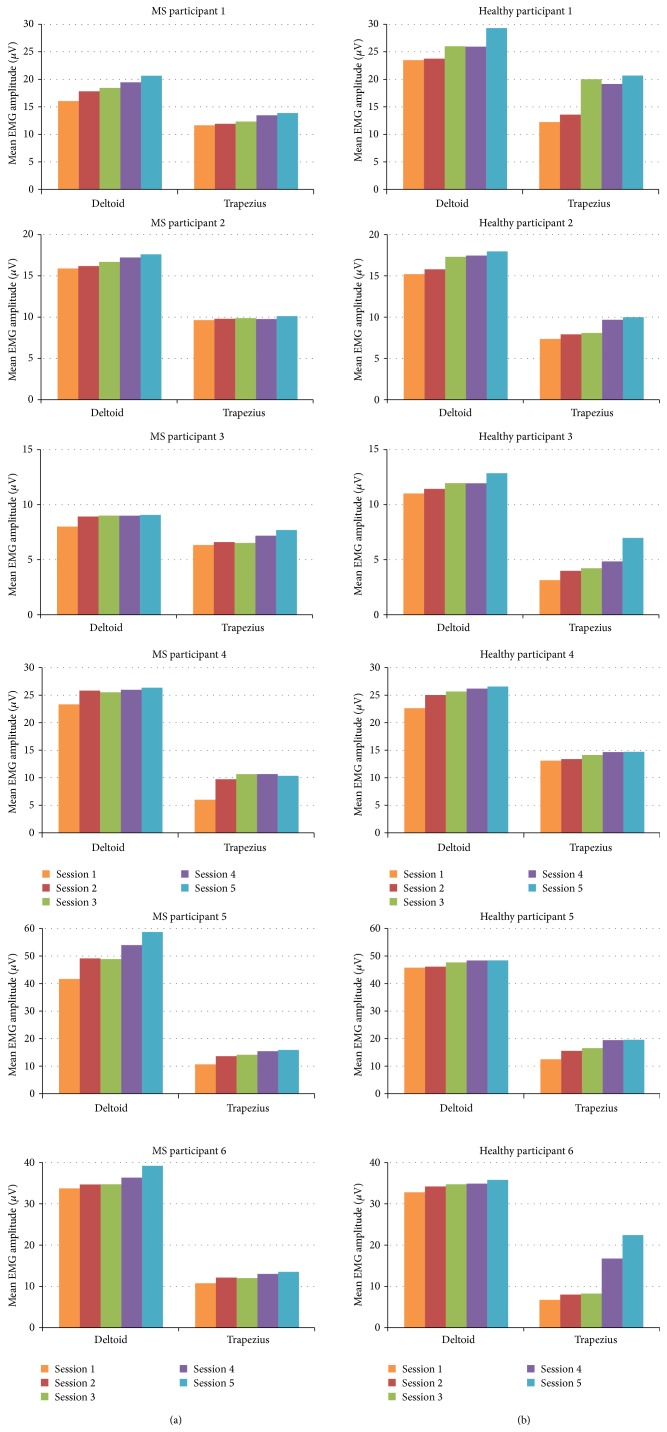
Mean EMG amplitudes of the anterior deltoid and lower trapezius muscle over the 5 training bouts (3′ each): MS participants (a) and healthy participants (b).

Figure 5 illustrates the increase in mean EMG amplitudes in the deltoid and trapezius muscles of MS and healthy participants over the course of five training bouts. For all participants, we observed an increase of EMG amplitudes in both the anterior deltoid and lower trapezius muscles during the training. It can be observed that the mean EMG amplitudes measured on the deltoid muscles were higher compared to the mean EMG amplitudes of the trapezius muscles. This was expected due to the role of the deltoid muscle as the agonist muscle or prime mover in performing lifting movements.

When comparing the mean EMG amplitudes between the first and last training bout, the increments of the deltoid muscles showed an average of 20.29% for MS participants and 15.25% for healthy participants. For both MS patients and healthy participants, Wilcoxon Signed-Rank tests showed that there is a significant increase of mean EMG amplitudes in the deltoid muscles between the first and last bout (*Z* = −2.201, *P* < 0.05).

These findings informed us that the development of muscle fatigue has been observed in both the anterior deltoid and lower trapezius muscles for MS patients and healthy participants. We found an increasing pattern of mean EMG amplitudes measured during the experiment, with a quite significant increase of the mean EMG amplitudes between the first and last training bout of the experiment.

### 3.3. Participant's Subjective Perception of Fatigue

**Figure 6:**
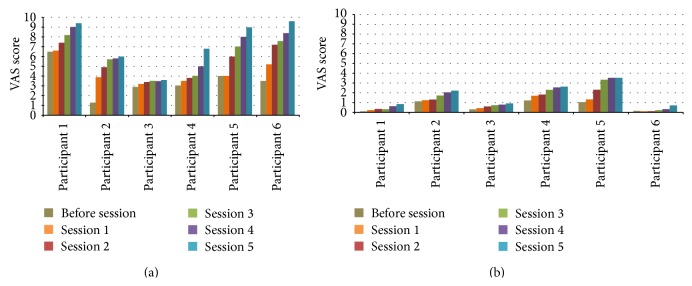
Participant's subjective perception on muscle fatigue over the 5 training bouts (3′ each): MS participants (a) and healthy participants (b).

We acquired participants' subjective responses on how they perceived the level of fatigue or tiredness on their arm.  Figure 6 gives an overview of the VAS scores for both MS and healthy participants over the five training bouts. A significantly higher VAS score among the MS participants can be observed compared to the healthy group indicating a higher perception of muscle fatigue by patients. It can be observed that the higher perception of fatigue is already present after as short as one training bout of 3 minutes. After the first training bout, initial VAS scores ranging from 3.2 to 6.6 (M = 4.40, SD = 1.28) were gathered from the MS participants while the scores from the healthy participants were ranging from 0.1 to 1.7 (M = 0.82, SD = 0.67). We can notice a similar increasing trend of VAS scores across the five training bouts in both groups of participants. At the end of the experiment, the last VAS scores were ranging from 3.6 to 9.6 (M = 7.40, SD = 2.37) for the MS participants and 0.7 to 3.5 (M = 1.78, SD = 1.16) for the healthy participants.

For both MS patients and healthy participants, Wilcoxon Signed-Rank tests showed that there is a significant increase in VAS scores between the first and last bout (*Z* = −2.201, *P* < 0.05). Mann-Whitney tests confirmed the significant difference of VAS scores between MS patients and healthy participants across all bouts (*P* < 0.05).

We can conclude that both MS patients and healthy participants perceived the level of fatigue in their arm to significantly increase over the training bouts. They rated to feel their arms to be more tired at the end of each training bout after performing the training exercise.

## 4. Discussion

In this pilot study, an experiment was carried out to investigate whether or not muscle fatigue develops in impaired arms of MS patients while performing a robot-mediated lifting exercise over a certain period of time. We have observed the phenomenon of muscle fatigue development during the training bouts since the increase in mean EMG amplitudes was shown in both the deltoid and trapezius muscles of participants, which are the main muscles used in the lifting movement. The demonstration of abnormal muscle fatigue in MS patients was supported by an observation that their training performance (game score) decreased while subjective perception of muscular fatigue increased to a large extent during the training session. If the experiment would have continued for a longer period, it would be most probable that the muscle fatigue experienced by the participants would escalate. When this happens, participants would begin to feel difficulties in moving their arms to perform lifting movements and as a result a more pronounced decrease of performance can be expected. These findings were observed in both MS patients with muscle weakness as those with relatively preserved muscle strength. Research on the relation between muscle weakness and fatigability has shown inconsistent results [19–21] suggesting that decreased muscle strength and abnormal muscle fatigue are different phenomena.

The present findings indicate the difficulty to effectively apply intensive training programs in MS, although it is considered important to involve patients in a high load of active and repetitive movements in order to stimulate neuroplasticity [35]. The amount of strength needed to perform the movements is not the most important consideration in this case; it is considered more important that MS patients are enabled to keep on training for a longer period of time.

We have considered the necessity in assisting the patients to perform active and repetitive movements in their training. The study showed that the muscle fatigue decreased their performance level. Therefore, aiming to optimize training performance of MS patients, we propose adaptivity to be integrated in their robot-mediated rehabilitation training. It is our intention to integrate adaptivity in robot-assisted upper limb rehabilitation for MS based on the detected muscle fatigue experienced by them during training. We can utilize the information on muscle fatigue developing in MS patients as real-time adaptivity triggers during training. Adaptivity can be realized, for example, by automatically giving assistance (antigravity support) to patients during training whenever needed (i.e., when the muscle fatigue develops). A previous study showed that MS patients obtain a larger 3D range of movement when applying automatically defined antigravity support based on a sustained arm position [16]. In another study, it was shown that MS patients obtained functional improvement after training with an electromechanical device providing antigravity support [14]. The idea of providing assistance in the form of antigravity support automatically during the training, thus not only determined once at the beginning or manually adapting, can be realized to help MS patients perform the exercise and sustain the training endurance.

In previous studies, we have investigated the possibility of providing adaptivity in the rehabilitation training exercises developed within I-TRAVLE [31, 36]. Adaptivity was provided by automatically and dynamically adjusting the difficulty of the exercises according to the patient's performance and training progress to avoid boredom, provide suitable challenge, and minimize the therapist's involvement. The user studies with nine MS patients showed that adaptive difficulty has delivered an adaptive personalized training to each MS patient according to his/her own individual training progress. Patients and the therapist have appreciated the automatic adaptation of difficulty levels and considered it to provide more variety in the training and also give the patients more freedom to train on their own without any interference from the therapist to manually adjust the exercise parameters. Another type of adaptation is automatically adjusting the training platform (virtual environment) parameters. For instance, normally a predetermined value of movement amplitude is required to perform a certain lifting movement. When the muscle fatigue develops, the required movement amplitude can be reduced to half of the normal value so that patients require less effort to perform the movement but their performance level is maintained. Further investigation is needed to enable defining an accurate adaptation algorithm for integrating these adaptations into the training of MS patients and also to investigate whether or not adaptivity helps to foresee an optimal training performance with more likelihood of sustained training intensity for MS patients.

In comparison to other studies related to measuring upper limb muscle fatigue in MS, our study applied a prolonged performance protocol that investigated the development of muscle fatigue in a longer period of training (15 minutes). This is clearly longer and more functional than some other studies with a very short measurement period (0.5–1 minute) which primarily aimed to calculate fatigue indexes [18, 19]. However, we acknowledge some limitations in the conduct of this study and interpretation of the results. First, this pilot study included only a small sample of participants (6 persons with MS and 6 healthy persons) so further study with a larger sample of participants is advised. In terms of game performance related to increased muscular effort during the training, the study showed a contradicting finding for healthy participants compared to patients. We were unable to see a decline in game performance during training, which we had expected to occur. This might be due to a learning effect in playing the game in the healthy participants which was however not found for patients. It may also be that the standard weight administered in healthy controls was relatively not heavy enough to elicit muscular fatigue in contrast to patients who may have been relatively more challenged in muscular efforts given the presence of muscle weakness. However, also the healthy controls had indicated an increase in subjective muscular fatigue in their arm. It could also be that the contradicting performance findings relate to motivational aspects with competitive attitude being greater in healthy controls, which were mainly therapists of the clinical rehabilitation center. We presume that a heavier lifting task with a longer period of training may eventually result in a performance decrease. Finally, it is a limitation that we have not measured the quality of motor performance and the potential occurrence of compensatory trunk and shoulder movements. These would have been secondary indicators of the development of muscular fatigue.

## 5. Conclusions

In summary, our findings in this study have provided preliminary evidence that muscle fatigue developed in persons with MS with impaired arm function during a single robot-assisted upper limb rehabilitation training session. These findings lead us to proposing adaptivity, based on the detection of muscle fatigue, to be integrated in the robot-mediated training program. Through adaptivity, sustained training intensity and optimized training performance are likely to be achieved for persons with MS.

## Figures and Tables

**Table 1:** Personal and clinical information of MS participants in the experiment.

MS participant	Personal information			Clinical characteristics		
	Gender	Age (years)	Diagnosis (years)	MI	BFM-prox.	BFM-dist.
MS1	Male	45	6	84	35	24
MS2	Male	57	26	84	39	24
MS3	Male	66	36	50	17	13
MS4	Female	63	17	91	41	24
MS5	Male	56	6	76	37	21
MS6	Male	62	6	70	36	24

MI: motricity index (best score = 100), BFM-prox.: Brunnstrom Fugl-Meyer proximal score (best score = 66), and BFM-dist.: Brunnstrom Fugl-Meyer distal score (best score = 66).
